# Long term safety and efficacy of the Yukon Choice Flex sirolimus-eluting coronary stent-a real-world data from India

**DOI:** 10.1016/j.ihj.2021.09.006

**Published:** 2021-09-10

**Authors:** Basant Kumar, Sourabh Agstam, Jyothi Vijay, Akash Batta

**Affiliations:** a Department of Cardiology, Advanced Cardiac Centre, Post Graduate Institute of Medical Education & Research, Chandigarh, 160012, India; b Department of Cardiology, Vardhman Mahavir Medical College, New Delhi, 110029, India; c Department of Cardiology, Sree Chitra Tirunal Institute of Medical Sciences and Technology, Trivandrum, Kerala 695011, India

**Keywords:** Drug-eluting stent, Yukon choice flex, Stent thrombosis, In-stent restenosis, YCF, Yukon Choice Flex, ACS, Acute coronary syndrome, STEMI, ST-elevation myocardial infarction, NSTEMI, Non-ST-elevation myocardial infarction, PCI, Percutaneous coronary intervention, DES, Drug-eluting stent, BMS, Bare metal stents

## Abstract

In-stent restenosis and stent thrombosis are the major concerns while choosing a coronary stent. This single-centre, retrospective study evaluated the one and three-year clinical outcomes following implantation of Yukon Choice Flex (YCF) sirolimus-eluting stent. A total of 168 consecutive patients with 217 lesions underwent stenting with YCF stent. The presentation was with acute coronary syndrome in 158 (94%) patients. At 3 years, 9 (5.3%) patients died due to cardiac cause. Myocardial infarction, and definite stent thrombosis occurred in 10 (6%) and 4 (2.4%) patients respectively. Redo stenting and coronary artery bypass surgery was performed in 3 (1.8%) and 1 (0.6%) patient respectively. The use of YCF sirolimus eluting stent was associated with a favourable safety and efficacy profile at one and three-years of follow-up in a high-risk population.

## 1 Introduction

Coronary revascularisation and primary angioplasty is standard of care in the setting of myocardial infarction.^1^ Despite ongoing improvements in stent design and antiplatelet drugs, concerns about stent thrombosis and in-stent restenosis continues to be an important aspect while contemplating an optimal revascularization strategy. Stent thrombosis refers to thrombotic occlusion of the stent and is associated with higher morbidity and mortality.^2^ In-stent restenosis is another limitation of percutaneous interventions leading to target lesion failure and recurrent interventions.^3^

In the last two decades, there has been tremendous improvement in stent design, implant technology and type from bare-metal stents (BMS) to various generations of drug-eluting stents (DES) to reduce the incidence of stent thrombosis and in-stent restenosis.

This study attempted to find out the real-world experience with sirolimus-eluting biodegradable polymer-based Yukon Choice Flex (YCF) (*Translumina Therapeutics, Dehradoon, India*) stent in a population predominantly consisting of an acute coronary syndrome (ACS).

## 2 Methods

This was a single-centre, retrospective, observational study carried out in a tertiary care centre in Northern India. A total of 168 consecutive patients with ACS or chronic coronary syndrome, who underwent percutaneous coronary intervention (PCI) with YCF stent from November 2015 till February 2017 were enrolled. Patients who died before PCI or had significant renal dysfunction or sepsis, severe thrombocytopenia or coagulation abnormalities, those who had a delayed presentation with refractory left ventricular failure (LVF) or refractory cardiogenic shock in whom PCI could not be performed, were not enrolled. The study aimed at studying the efficacy and outcomes of patients who underwent PCI with YCF stent at 1 and 3 years.

The study protocol conformed to the ethical guidelines of the declaration of Helsinki and was reviewed and cleared by the institute's ethical committee.

Clinical diagnosis included 88 (52.4%) patients with ST-segment elevation myocardial infarction (STEMI), 70 (41.6%) patients with non–ST-segment elevation ACS [including non-ST elevation myocardial infarction (NSTEMI) in 42 (25%) patients and unstable angina in 28 (16.6%) patients], and 10 (6%) patients with chronic coronary syndromes (Table 1). The culprit lesion was identified by the combination of electrocardiographic changes, echocardiogram findings, and angiographic lesion morphology.

**Table 1:** Baseline characteristics of study population (*n* = 168).

Variables	n, (%)
Patients	168
Age, years	56.4 ± 12.68
Sex, n (%)	
Males	146 (85.9)
Females	22 (14.1)
Mean Body Mass Index (BMI) (kg/m^2^)	24.5 ± 4.92
**Risk Factors, n (%)**	
Hypertension	55 (32.4)
Diabetes mellitus	31 (18.2)
Smoking	63 (37.1)
Dyslipidemia	70 (41.6)
Family history of coronary artery disease	37 (21.8)
Ejection fraction (%)	46.3 ± 9.21
Hemoglobin (g/dL)	13.1 ± 2.02
Creatinine (mg/dL)	0.8 ± 0.3
**Diagnosis, n (%)**	
STEMI	88 (52.4)
NSTEMI	42 (25)
USA	28 (16.6)
Stable Angina	10 (6.0)

All values are presented as the n (%). Continuous variables were presented as mean ± S.D. BMI, body mass index; CAD, coronary artery disease; NSTEMI, Non-ST elevation myocardial infarction, STEMI, ST-elevation myocardial infarction; USA, unstable angina.

Statistical analysis was performed with Statistical Package for the Social Sciences version 26 (SPSS Inc., Chicago, IL, United States).

## 3 Results

A total 217 YCF stents were deployed in 168 patients. The mean age of the participants were 56.4 ± 12.68 years. The study population were predominantly male (85.9%). The presentation was with ACS in 158 (94%) of study subjects. The baseline characteristics of patients were summarized in Table 1.

The baseline angiographic characteristics were tabulated in Table 2. The left anterior descending artery was the commonest artery intervened. Majority of the patients had single-vessel disease, followed by triple vessel disease and double vessel disease. Around 1/5th of the lesions were ostial in location. The mean stented length was 28.34 ± 8.27 mm and the mean stent diameter was 3.01 ± 0.43 mm. PCI was successful in all lesions and the immediate angiographic outcomes were excellent in all cases.

**Table 2:** Baseline angiographic parameters.

Vessel stented, n (%)	217
Left Main	4 (1.8)
Left Anterior Descending	110 (50.7)
Left Circumflex	47 (21.7)
Right Coronary Artery	53 (24.5)
Ramus	3 (1.3)
Vessel status, n (%)	168
Single vessel disease	88 (52.4)
Double vessel disease	28 (16.7)
Triple vessel disease	52 (30.9)
Lesion location, n (%)	217
Ostial stenosis	40 (18.4)
Proximal segment	64 (29.5)
Mid segment	85 (39.2)
Distal segment	15 (6.9)
Bifurcation lesions	13 (5.9)
Stents	217
Mean Stent length, mm	28.34 ± 8.27
Mean Stent diameter, mm	3.01 ± 0.43

Clinical follow up was performed for all patients for three years. At the end of 3 years, a total of 12 (7.1%) deaths occurred, out of which 9 (5.3%) patients died due to presumed cardiac cause. Other outcomes assessed at the end of 3 years was myocardial infraction in 10 (6%) patients, definite stent thrombosis in 4 (2.4%) patients and target lesion revascularization was needed in 2 (1.2%) patients. Individual outcomes at the end of 1 year and from 1 to 3 years are summarized in Table 3.

**Table 3:** Clinical follow-up data at 1 year and 3-year (*n* = 168).

Duration	1- year, n (%)	1-3-year, n (%)
Death Total (*n* = 12, 7.1%)	7 (4.2)	5 (2.9)
Death due to cardiac cause (*n* = 9, 5.3%)	5 (2.9)	4 (2.4)
Myocardial infarction (*n* = 10, 6%)	3 (1.8)	7 (4.2)
Definite stent thrombosis (Reinfarction) (*n* = 4, 2.4%)	1 (0.6)	3 (1.8)
TLR (*n* = 2, 1.2%)	1 (0.6)	1 (0.6)
Redo -PCI (*n* = 3, 1.8%)	0	3 (1.8)
CABG (*n* = 1, 0.6%)	1 (0.6)	0

All values are presented as the n (%). CABG, coronary artery bypass grafting; MACEs, major adverse cardiac events; TLR, target lesion revascularization; PCI, percutaneous coronary intervention.

Redo PCI was done in 3 patients. Coronary artery bypass graft surgery was done in 1 patient during the study period.

## 4 Discussion

PCI is the revascularisation modality of choice in most patients with ACS and suitable coronary anatomy. The odds of the development of stent failure in the form of in-stent restenosis and stent thrombosis are important considerations while selecting a stent.^2^^,^^3^ Hence there have been numerous attempts to use the best possible stent technology. YCF stent is a commonly used sirolimus-eluting DES with a biodegradable polymer and strut thickness of 87 μm. Other advantages are microporous surface and abluminal coating.^4^^,^^5^

Even though the debate over the efficacy and safety of DES versus BMS is continuing; contemporary data suggest that the benefits of DES outweigh the risks compared with BMS mirroring the current increase in DES usage.^6^ The use of first generation DES is independently associated with stent thrombosis likely secondary to the limited flexibility of the first-generation DES, or endothelial damage at the time of stent deployment.^7^ Hence, newer generation stents with improvement in stent design have been developed.

Our study population consisted predominantly of ACS patients. More than half of the patients with ACS had STEMI which was similar to other studies from India and west including the INTERHEART study.^8^^,^^9^ ACS patients are more likely to develop stent thrombosis than stable coronary artery disease and this portends a poorer outcome.^10^ The predominantly male population in the study could be attributed to the increased prevalence of ACS in males along with the skewed risk factor distribution like smoking or underutilization of health care resources by women in this region. The prevalence of dyslipidaemia, smoking and hypertension were comparable to other studies from this region.^11^

In an observational study of YCF stent by Xhepa et al, the rates of death, myocardial infarction, definite stent thrombosis and ischemia-driven target lesion revascularization were 2.4%, 1.9%, 0.3% and 11.3% respectively at 1 year.^12^ We observed a lower rate of myocardial infarction (1.4% versus 1.9%) at 1 year. This was even though their study population comprised of 40.9% patients with ACS compared to 94% patients in our study. The current study showed comparable efficacy of the YCF stent in a high-risk population. The better outcomes in our study could be due to the rigorous follow-up, and ensuring adherence to medications and healthy life-style in our population.

We would like to reiterate the fact that the study aimed to study the efficacy of YCF stent and the intermediate and long-term outcomes of patient undergoing PCI with YCF. It was not designed to look at outcomes of ACS patients as such. Still the lower 1 year mortality at 4.2% in our study can be explained by the relatively younger patient powith mean patient age of 56.4 ± 12.68 years. Much lesser patients had diabetes compared to other larger studies and the mean ejection fraction was higher at 46.3 ± 9.21% compared to prior studies.^13^^,^^14^ All our patients had undergone PCI with DES. Studies have shown that younger patient age, a higher ejection fraction and PCI are all independently associated with improved long term outcomes.^13^, ^14^, ^15^

A study similar to ours by Liu et al which enrolled patients of STEMI and NSTEMI undergoing PCI with DES had an overall mortality rate of 5.6% at 5 years and was comparable to our data of 7.1% mortality at 3 years.^16^ The similarity to this study can be explained on the basis that both studies recruited only patients undergoing PCI and not the entire ACS cohort. However, we accept that our study and the study by Liu et al are not ideal to comment on long term outcomes after ACS, however they indeed demonstrate the efficacy and safety of the DES in this population.

The major limitations of our study was the absence of routine angiographic follow up and a scarce use of intravascular imaging for PCI optimization.

The mean left ventricle ejection fraction in our study was 46.3 ± 9.21%, which was higher for a cohort predominantly comprising of an ACS population. The sickest of patients who either died before PCI could be performed or had significant renal dysfunction or sepsis, severe thrombocytopenia or coagulation abnormalities or had a delayed presentation with refractory LVF or refractory cardiogenic shock in whom PCI could not be performed, were not enrolled. One would expect these patients to be having a lower LVEF compared to patients undergoing PCI in our study. As a result, selection bias cannot be ruled out.

This study provides 3-year clinical outcome data of Sirolimus-eluting Yukon choice flex stent in a real-world setting and highlights the fact that improved stent design technology will lead to comparable clinical outcomes.

In conclusion, the use of YCF sirolimus eluting stent was associated with a favourable safety and efficacy profile at one and three-years of follow-up in a high-risk population.

## Declaration of competing interest

There is no conflict of interest amongst the authors in regards to the present study.
