# Supplementary material for: Long term safety and efficacy of the Yukon Choice Flex sirolimus-eluting coronary stent-a real-world data from India
Source: Indian Heart J. 2021 Sep 10;73(6):733–6. doi: 10.1016/j.ihj.2021.09.006 (PMC8642663; doi:10.1016/j.ihj.2021.09.006)
Supplement: Multimedia component 1 [file mmc1.docx]

**Long term safety and efficacy of the Yukon Choice Flex Sirolimus-eluting coronary stent- a real-world data from India**

**Abstract**

In-stent restenosis and stent thrombosis are the major concerns while choosing a coronary stent. This single-centre, retrospective study evaluated the one and three-year clinical outcomes following implantation of Yukon Choice Flex (YCF) sirolimus-eluting stent. A total of 168 consecutive patients with 217 lesions underwent stenting with YCF stent. The presentation was with acute coronary syndrome in 158 (94%) patients. At 3 years, 9 (5.3%) patients died due to cardiac cause. Myocardial infarction, and definite stent thrombosis occurred in 10 (6%) and 4 (2.4%) patients respectively. Redo stenting and coronary artery bypass surgery was performed in 3 (1.8%) and 1 (0.6%) patient respectively.

**Keywords;** Drug-eluting stent, Yukon choice flex, Stent thrombosis, In-stent restenosis.

**1. Introduction**

Coronary revascularisation and primary angioplasty is standard of care in the setting of myocardial infarction^1^. Despite ongoing improvements in stent design and antiplatelet drugs, concerns about stent thrombosis and stent restenosis continues to be an important aspect while contemplating an optimal revascularization strategy. Stent thrombosis refers to thrombotic occlusion of the stent and is associated with higher mortality and morbidity^2^. In-stent restenosis is another limitation of percutaneous interventions leading to target lesion failure and recurrent interventions^3^.

In the last two decades, there has been tremendous improvement in stent design, implant technology and type from bare-metal stents (BMS) to various generations of drug-eluting stents (DES) to reduce the incidence of stent thrombosis and in-stent restenosis.

This study attempted to find out the real-world experience with sirolimus-eluting biodegradable polymer-based Yukon Choice Flex (YCF) (*Translumina Therapeutics, Dehradoon, India*) stent in a population predominantly consisting of an acute coronary syndrome (ACS).

**2. Methods**

This was a single-centre, retrospective, observational study carried out in a tertiary care centre in Northern India. A total of 168 consecutive patients with ACS or chronic coronary syndrome, who underwent percutaneous coronary intervention (PCI) with YCF stent from November 2015 till February 2017 were enrolled. Patients who died before PCI or had significant renal dysfunction or sepsis, severe thrombocytopenia or coagulation abnormalities or had a delayed presentation with refractory left ventricular failure (LVF) or refractory cardiogenic shock in whom PCI could not be performed, were not enrolled. The study aimed at studying the efficacy and outcomes of patients who underwent PCI with YCF stent at 1 and 3 years.

The study protocol conformed to the ethical guidelines of the declaration of Helsinki and was reviewed and cleared by the institute’s ethical committee.

Clinical diagnosis included 88 (52.4%) patients with ST-segment elevation myocardial infarction (STEMI), 70 (41.6%) patients with non–ST-segment elevation ACS [including non-ST elevation myocardial infarction (NSTEMI) in 42 (25%) patients and unstable angina in 28 (16.6%) patients], and 10 (6%) patients with chronic coronary syndromes (Table 1). The culprit lesion was identified by the combination of electrocardiographic changes, echocardiogram findings, and angiographic lesion morphology.

Statistical analysis was performed with Statistical Package for the Social Sciences version 26 (SPSS Inc., Chicago, IL, United States).

**3. Results**

A total 217 YCF stents were deployed in 168 patients. The mean age of the participants were 56.4 + 12.68 years. The study population were predominantly male (85.9%). The presentation was with ACS in 158 (94%) of study subjects. The baseline characteristics of patients were summarized in table 1.

The baseline angiographic characteristics were tabulated in table 2. The left anterior descending artery was the commonest artery intervened. Majority of the patients had single-vessel disease, followed by triple vessel disease and double vessel disease. Around 1/5^th^ of the lesions were ostial in location. The mean stented length was 28.34 + 8.27 mm and the mean stent diameter was 3.01 + 0.43 mm. PCI was successful in all lesions and the immediate angiographic outcomes were excellent in all cases.

Clinical follow up was performed for all patients for three years. At the end of 3 years, a total of 12 (7.1%) deaths occurred, out of which 9 (5.3%) patients died due to presumed cardiac cause. Other outcomes assessed at the end of 3 years was myocardial infraction in 10 (6%) patients, definite stent thrombosis in 4 (2.4%) patients and target lesion revascularization was needed in 2 (1.2%) patients. Individual outcomes at the end of 1 year and from 1-3 years are summarized in table 3.

Redo PCI was done in 3 patients. Coronary artery bypass graft surgery was done in 1 patient during the study period.

**4. Discussion**

PCI is the revascularisation modality of choice in most patients with ACS and suitable coronary anatomy. The odds of the development of stent failure in the form of in-stent restenosis and stent thrombosis are important considerations while selecting a stent^2,3^. Hence there have been numerous attempts to use the best possible stent technology. YCF stent is a commonly used sirolimus-eluting DES with a biodegradable polymer and strut thickness of 87µm. Other advantages are microporous surface and abluminal coating^4,5^.

Even though the debate over the efficacy and safety of DES versus BMS is continuing; contemporary data suggest that the benefits of DES outweigh the risks compared with BMS mirroring the current increase in DES usage^6^. The use of first generation DES is independently associated with stent thrombosis likely secondary to the limited flexibility of the first-generation DES, or endothelial damage at the time of stent deployment^7^. Hence, newer generation stents with improvement in stent design have been developed.

Our study population consisted predominantly of ACS patients. More than half of the patients with ACS had STEMI which was similar to other studies from India and west including the INTERHEART study^8,9^. ACS patients are more likely to develop stent thrombosis than stable coronary artery disease and this portends a poorer outcome^10^. The predominantly male population in the study could be attributed to the increased prevalence of ACS in males along with the skewed risk factor distribution like smoking or underutilization of health care resources by women in this region. The prevalence of dyslipidaemia, smoking and hypertension were comparable to other studies from this region^11^.

In an observational study of YCF stent by Xhepa et al, the rates of death, myocardial infarction, definite stent thrombosis and ischemia-driven target lesion revascularization were 2.4%, 1.9%, 0.3% and 11.3% respectively at 1 year^12^. We observed a lower rate of myocardial infarction (1.4% versus 1.9%) at 1 year. This was even though their study population comprised of 40.9% patients with ACS compared to 94% patients in our study. The current study showed comparable efficacy of the YCF stent in a high-risk population. The better outcomes in our study could be due to the rigorous follow-up, and ensuring adherence to medications and healthy life-style in our population.

We would like to reiterate the fact that the study aimed to study the efficacy of YCF stent and the short and intermediate-term outcomes of patient undergoing PCI with YCF. It was not designed to look at outcomes of ACS patients as such. Still the lower 1 year mortality at 4.2% in our study can be explained by the relatively younger with mean patient age of 56.4 + 12.68 years. Much lesser patients had diabetes compared to other larger studies and the mean ejection fraction was higher at 46.3 + 9.21% compared to prior studies^13,14^. All our patients had undergone PCI with DES. Studies have shown that younger patient age, a higher ejection fraction and PCI are all independently associated with improved long term outcomes^13–15^.

A study similar to ours by Liu et al. which enrolled patients of STEMI and NSTEMI undergoing PCI with DES had an overall mortality rate of 5.6% at 5 years and was comparable to our data of 7.1% mortality at 3 years^16^. The similarity to this study can be explained on the basis that both studies recruited only patients undergoing PCI and not the entire ACS cohort. However, we accept that our study and the study by Liu et al. are not ideal to comment on long term outcomes after ACS, however they indeed demonstrate the efficacy and safety of the DES in this population.

The major limitations of our study was the absence of routine angiographic follow up and a scarce use of intravascular imaging for PCI optimization.

The mean left ventricle ejection fraction in our study was 46.3 + 9.21%, which was higher for a cohort predominantly comprising of an ACS population. The sickest of patients who either died before PCI could be performed or had significant renal dysfunction or sepsis, severe thrombocytopenia or coagulation abnormalities or had a delayed presentation with refractory LVF or refractory cardiogenic shock in whom PCI could not be performed, were not enrolled. One would expect these patients to be having a lower LVEF compared to patients undergoing PCI in our study. As a result, selection bias cannot be ruled out.

This study provides 3-year clinical outcome data of Sirolimus-eluting Yukon choice flex stent in a real-world setting and highlights the fact that improved stent design technology will lead to comparable clinical outcomes.

| In conclusion, the use of YCF sirolimus eluting stent was associated with a favourable safety and efficacy profile at one and three-years of follow-up in a high-risk population. |
| --- |

**Key message:**

**Acknowledgement:** No additional contribution by any other person.

**Conflict of Interests:** There is no conflict of interest amongst the authors in regards to the present study.

**Author's contribution**: All the authors were involved in [1] substantial contributions to research design, acquisition, analysis, or interpretation of data; [2] drafting the paper or revising it critically; [3] approval of the submitted and final versions.

**References:**

1. Neumann F-J, Sousa-Uva M, Ahlsson A, et al. 2018 ESC/EACTS Guidelines on myocardial revascularization. *Eur Heart J*. 2019;40(2):87-165.

2. Claessen BE, Henriques JP, Jaffer FA, Mehran R, Piek JJ, Dangas GD. Stent thrombosis: a clinical perspective. *JACC Cardiovasc Interv*. 2014;7(10):1081-1092.

3. Buccheri D, Piraino D, Andolina G, Cortese B. Understanding and managing in-stent restenosis: a review of clinical data, from pathogenesis to treatment. *J Thorac Dis*. 2016;8(10):E1150.

4. Mehilli J, Kastrati A, Wessely R, et al. CLINICAL PERSPECTIVE. *Circulation*. 2006;113(2):273-279.

5. Wessely R, Hausleiter J, Michaelis C, et al. Inhibition of neointima formation by a novel drug-eluting stent system that allows for dose-adjustable, multiple, and on-site stent coating. *Arterioscler Thromb Vasc Biol*. 2005;25(4):748-753.

6. Sung S-H, Chen T-C, Cheng H-M, Lee J-C, Lang H-C, Chen C-H. Comparison of clinical outcomes in patients undergoing coronary intervention with drug-eluting stents or bare-metal stents: a nationwide population study. *Acta Cardiol Sin*. 2017;33(1):10.

7. Tada T, Byrne RA, Simunovic I, et al. Risk of stent thrombosis among bare-metal stents, first-generation drug-eluting stents, and second-generation drug-eluting stents: results from a registry of 18,334 patients. *JACC Cardiovasc Interv*. 2013;6(12):1267-1274.

8. Misiriya K, Sudhayakumar N, Khadar SA, George R, Jayaprakasht V, Pappachan JM. The clinical spectrum of acute coronary syndromes: experience from a major center in Kerala. *J Assoc Physicians India*. 2009;57:377-383.

9. Yusuf S, Hawken S, Ôunpuu S, et al. Effect of potentially modifiable risk factors associated with myocardial infarction in 52 countries (the INTERHEART study): case-control study. *The lancet*. 2004;364(9438):937-952.

10. Xu Y, Qu X, Fang W, Chen H. Prevalence, Correlation and Clinical Outcome of Intra‐Procedural Stent Thrombosis in Patients Undergoing Primary Percutaneous Coronary Intervention for Acute Coronary Syndrome. *J Intervent Cardiol*. 2013;26(3):215-220.

11. Wander G, Khurana S, Gulati R, Sachar R, Gupta R. Epidemiology of Coronary Heart Disease in a Rural Punjab Population-Prevalence and Correlation. *Indian Heart J*. 1994;46(6):319-323.

12. Xhepa E, Tada T, Cassese S, et al. Safety and efficacy of the Yukon Choice Flex sirolimus-eluting coronary stent in an all-comers population cohort. *Indian Heart J*. 2014;66(3):345-349.

13. Santos IS, Goulart AC, Brandão RM, et al. One-year mortality after an acute coronary event and its clinical predictors: the ERICO study. *Arq Bras Cardiol*. 2015;105:53-64.

14. Chen X, Barywani SB, Sigurjonsdottir R, Fu M. Improved short and long term survival associated with percutaneous coronary intervention in the elderly patients with acute coronary syndrome. *BMC Geriatr*. 2018;18(1):1-9.

15. Pocock S, Bueno H, Licour M, et al. Predictors of one-year mortality at hospital discharge after acute coronary syndromes: A new risk score from the EPICOR (long-tErm follow uP of antithrombotic management patterns In acute CORonary syndrome patients) study. *Eur Heart J Acute Cardiovasc Care*. 2015;4(6):509-517.

16. Liu H, Jin Z, Yang S, et al. Five-year outcomes of ST-elevation myocardial infarction versus non-ST-elevation acute coronary syndrome treated with biodegradable polymer-coated sirolimus-eluting stents: Insights from the CREATE trial. *J Cardiol*. 2017;69(1):149-155.
